# Evidence for Overlapping and Distinct Biological Activities and Transcriptional Targets Triggered by Fibroblast Growth Factor Receptor 2b Signaling between Mid- and Early Pseudoglandular Stages of Mouse Lung Development

**DOI:** 10.3390/cells9051274

**Published:** 2020-05-21

**Authors:** Matthew R. Jones, Arun Lingampally, Jin Wu, Jamschid Sedighi, Negah Ahmadvand, Jochen Wilhelm, Ana Ivonne Vazquez-Armendariz, Susanne Herold, Chengshui Chen, Jin-San Zhang, Saverio Bellusci, Cho-Ming Chao

**Affiliations:** 1 Key Laboratory of Interventional Pulmonology of Zhejiang Province, Department of Pulmonary and Critical Care Medicine, The First Affiliated Hospital of Wenzhou Medical University, Wenzhou 325035, China; matthew.jones@innere.med.uni-giessen.de (M.R.J.); Arun.K.Lingampally@med.uni-giessen.de (A.L.); Negah.Ahmadvand@innere.med.uni-giessen.de (N.A.); chenchengshui@wmu.edu.cn (C.C.); Zhang_jinsan@wmu.edu.cn (J.-S.Z.); 2 Cardio-Pulmonary Institute, Institute of Lung Health and Department of Pulmonary and Critical Care Medicine and Infectious Diseases, Universities of Giessen and Marburg Lung Center (UGMLC), Member of the German Center for Lung Research (DZL), Justus-Liebig University Giessen, 35392 Giessen, Germany; jamschid.sedighi@gmail.com (J.S.); Jochen.Wilhelm@patho.med.uni-giessen.de (J.W.); Ana.I.Vazquez-Armendariz@innere.med.uni-giessen.de (A.I.V.-A.); Susanne.Herold@innere.med.uni-giessen.de (S.H.); 3 International Collaborative Center on Growth Factor Research, Life Science Institute, Wenzhou University, Wenzhou 325035, China; cugin1519@163.com; 4 Division of General Pediatrics and Neonatology, University Children’s Hospital Gießen,Justus-Liebig-University, 35392 Giessen, Germany

**Keywords:** lung development, Fgf, Fgfr2b, pseudoglandular

## Abstract

Branching morphogenesis is the basic developmental mode common to organs such as the lungs that undergo a process of ramification from a rudimentary tree. However, the precise molecular and cellular bases underlying the formation of branching organs are still unclear. As inactivation of fibroblast growth factor receptor 2b (Fgfr2b) signaling during early development leads to lung agenesis, thereby preventing the analysis of this pathway at later developmental stages, we used transgenic mice to induce expression of a soluble form of Fgfr2b to inactivate Fgfr2b ligands at embryonic day (E) 14.5, corresponding to the mid-pseudoglandular stage of lung development. We identified an Fgfr2b signaling signature comprised of 46 genes enriched in the epithelium, some of which were common to, but most of them distinct from, the previously identified Fgfr2b signaling signature at E12.5. Our results indicate that Fgfr2b signaling at E14.5 controls mostly proliferation and alveolar type 2 cell (AT2) differentiation. In addition, inhibition of Fgfr2b signaling at E14.5 leads to morphological and cellular impairment at E18.5, with defective alveolar lineage formation. Further studies will have to be conducted to elucidate the role of Fgfr2b signaling at successive stages (canalicular/saccular/alveolar) of lung development as well as during homeostasis and regeneration and repair after injury.

## 1. Introduction

In spite of intense research efforts, the precise molecular and cellular bases underlying the formation of branching organs are still unclear. A process called branching morphogenesis is the basic developmental mode common to organs that undergo a process of ramification from an original rudimentary structure or primary tree. We and others have previously reported mechanistic studies aiming to elucidate how the embryonic lung develops [1,2]. The development of the mouse respiratory system begins around embryonic day (E) 9.5 with the evagination of two primary lung buds from a specialized domain of *Nkx2-1* expression in the ventral anterior foregut endoderm. Also, from this specialized domain, and just anterior to the primary buds, the foregut tube separates to form the future trachea and esophagus. Soon after the initial formation, both the trachea and lung buds elongate, while the highly stereotyped process of branching morphogenesis commences in the latter. By E12.5 the four main lobes in the right lung, and the one main lobe in the left, have been clearly established, and by E16.5 the airway epithelium has formed a highly branched and stereotyped tree-like structure ending in thousands of terminal tips [3].

This period, from E9.5-E16.5 in mice, is commonly referred to as the pseudoglandular stage of lung development, while some authors distinguish between a separate embryonic stage (E9.5–E12) before the pseudoglandular stage (E12.5–E16.5) [4]. Following the pseudoglandular stage is the canalicular stage (E16.5–E17.5), the saccular stage (E17.5-postnatal (PN) day 5), and the alveolar stage (PN5–PN28) [5,6,7]. 

Major signaling molecules mediating the complex mesenchymal-epithelial crosstalk initiating and regulating early lung development include bone morphogenetic factors (Bmps) and transforming growth factor beta (Tgfβ), Wnts, retinoic acid (RA), sonic hedgehog (Shh), and fibroblast growth factors (Fgfs) [6]. Of chief importance, especially for lung bud initiation and early branching morphogenesis, is Fgf10 signaling; the absence of either *Fgf10* or its cognate receptor *Fgfr2b* leads to complete lung agenesis, as well as impaired development of other branching organs such as the pancreas, prostate, mammary glands, and salivary and lacrimal glands [8,9,10].

Supporting Fgf10’s role in lung branching, we previously reported that *Fgf10* is strongly expressed in the lung mesenchyme adjacent to nascent epithelial buds [11]. These distal epithelial cells, thought to be the target of Fgf10 signaling during the branching process, are positive for the transcription factors Sox9 and Id2 and have been reported as epithelial multipotent stem cells capable of giving rise to both alveolar and bronchiolar lineages [12]. Using a transgenic model allowing the inducible expression of a mutated dominant negative form of the main Fgf10 receptor Fgfr2b, we previously published the role of Fgf10 in these cells at E12.5 and concluded that Fgf10 was necessary to regulate cell–cell and cell–extracellular matrix interactions during morphogensis, beta-catenin signaling, as well as the differentiation status of these cells [13]. Interestingly, no effects on proliferation or cell survival were detected in our E12.5 experiments, which assessed the regulation by Fgf10 sigalling after a 6- and 9-hour time-period. Furthermore, a comprehensive set of more than 40 genes downstream of Fgf10 signaling was also identified. Interestingly, this list contains genes which are markers of the differentiated alveolar epithelial cell type 2 (AT2), such as surfactant protein c (*Sftpc*), *Sftpa1*, *Sftpb*, ETS variant transcription factor 5 *(Etv5)*, as well as markers of the AT1 cells, such as homeodomain only protein x (*Hopx*) and *podoplanin* (*Pdpn*). These results suggest that Fgf signaling could indeed be active not only on multipotent epithelial progenitor cells but also on the subsequent progenitors of the future mature AT1 and AT2 cells. Supporting this idea, we previously reported that *Fgf10* hypomorphic pups, with around 30% *Fgf10* expression compared to normal pups, are born alive but die shortly after birth from a plethora of developmental defects, the main one being impaired lung development. E18.5 *Fgf10* hypomorphic lungs display impaired AT2 differentiation illustrated by decreased expression of Sftpb and Sftpc, as well as other defects, including abnormal formation of the alveolar myofibroblasts and aborted vascular development [14]. Interestingly, even though *Fgf10* heterozygous pups are born alive and can grow and reproduce normally without any obvious developmental defects, the corrrsponding 50% reduction in *Fgf10* expression leads to AT2 differentiation defects at birth and to a dramatic increase in the sensibility of the mutant pups to oxygen injury [15]. One limitation of the two studies mentioned above is that it is difficult to pinpoint the precise activity of Fgf10 in the lung, as the decrease in *Fgf10* expression is constitutive and therefore occurs from the very early stages of lung development. In these conditions, distinguishing Fgf10’s primary from secondary effects at different developmental stages is very challenging.

With these limitations in mind, here we report the findings of our continued investigation into the role of Fgfr2b signaling during the pseudoglandular stage of lung development. We chose E14.5 as our time point of inquiry, and refer to this point as mid-pseudoglandular lung development. We used a transgenic mouse model (*Rosa26^rtTA/rtTA^*; *Tg(tet(o)solFgfr2b/+*) to transiently or continuously inhibit Fgfr2b signaling at E14.5. This is a relatively robust model compared to another previously described soluble Fgfr2b system [16], which relies on a driver (*Sftpc^rtTA^*) with limited expression at this early timepoint. For transient inhibition, we administered a single intraperitoneal injection (IP) of doxycycline and harvested transgenic and littermate control lungs 9 h after inhibition. For continuous inhibition, we used doxycycline food and harvested the lungs at E18.5. With this approach, we were able to identify immediate transcriptional targets and biological activities of Fgfr2b signaling, as well as the role of this signaling pathway over time. Our results indicate a shifting in the biological activities of Fgfr2b signaling between early (E12.5) and mid (E14.5) pseudoglandular stages of mouse lung development, illustrating the need for further investigation of Fgfr2b signaling within and between different stages of lung development.

## 2. Materials and Methods 

### 2.1. Ethical Statement and Husbandry

Animal experiments and harvesting organs and tissues from wild type and mutant mice following euthanasia using pentobarbital were approved at Justus Liebig University Giessen by the federal authorities for animal research of the Regierungspraesidium Giessen, Hessen, Germany (Approved Protocol GI 20/10 Nr. G 84/2016).

All mice used to generate experimental and control embryos were housed in a specific pathogen-free (SPF) environment with free access to food and water. Up to five females were housed together, while males were housed singly. Females between 9–12 weeks of age were used to generate embryos. 

### 2.2. Mouse Model to Inhibit Fgfr2b Ligands

In vivo studies were conducted using a previously described and validated inducible dominant negative mouse model: *Rosa26^rtTA/rtTA^; Tg(tet(o)sFgfr2b)/+* (B6-Cg-Gt(ROSA)26Sor^tm1.1(rtTA,EGFP)Nagy^ Tg(tetO-Fgfr2b/lgh)1.3Jaw/sbel) [13,16,17]. In this model, a soluble form of *Fgfr2b* (*sFgfr2b*) is expressed after administration of doxycyline. The sFgfr2b is secreted from cells and acts to sequester Fgfr2b ligands, preventing them from binding and activating their endogenous receptors. Experimental [*Rosa26^rtTA/rtTA^; Tg(tet(o)sFgfr2b)/+*] and littermate control [*Rosa26^rtTA/rtTA^; +/+*] embryos were generated by crossing [*Rosa26^rtTA/rtTA^; Tg(tet(o)sFgfr2b)/+*] and [*Rosa26^rtTA/rtTA^; +/+*] animals. Doxycycline was administered via intraperitoneal injection (i.p.) or food to timed-pregnant females to conduct in vivo experiments, as previously described [13].

### 2.3. Embryonic Lung Dissection and Imaging

Following the sacrifice of experimental and control embryos, lungs were harvested and still images captured as previously described [18]. Lungs were then processed for RNA isolation or fixed in 4% PFA for subsequent paraffin embedding (see below).

### 2.4. DNA Isolation and PCR

DNA was isolated from the tails of E14.5 embryos. Gene-specific primers were used to detect the presence of *Rosa26^rtTA^* (wild type and transgene specific forward primer 5’-GAG TTC TCT GCT GCC TCC TG; wild type specific reverse primer 5’-CGA GGC GGA TAC AAG CAA TA; transgene specific reverse primer 5’-AAG ACC GCG AAG AGT TTG TC; expected product size of approximately 200 bp for the transgene and 322 bp for the wild type) and Tg(*tet(o)sFgfr2b)* (transgene specific forward primer 5’-CAG GCC AAC CAG TCT GCC TGG C; transgene specific reverse primer 5’-CGT CTG AGC TGT GTG CAC CTC C; expected product size of 310 bp). The PCR reaction mix was calculated for 20 µL per reaction, and included 10 µL 2 × Taq PCR Master Mix (Qiagen, Hilden, Germany), primers at a final concentration of 0.2 µM, RNase-free water, and up to 1 µg of genomic DNA. PCRs were performed in a C1000 Thermocycler (Bio-Rad, Feldkirchen, Germany). The cycling protocol to amplify *Rosa26^rtTA^* was as follows: initial denaturation at 94 °C for 3 min; 35 cycles of denaturation at 94 °C for 1 min, annealing at 63 °C for 1 min, and extension at 72 °C for 1.5 min; final extension at 72 °C for 5 min; and hold at 4 °C. The protocol to amplify Tg(*tet(o)sFgfr2b*) was as follows: initial denaturation at 93 °C for 4 mins; 40 cycles of denaturation at 95 °C for 30 s, annealing at 58 °C for 30 s, and extension at 72 °C for 30 s; final extension at 72 °C for 10 min; and hold at 4 °C. Capillary gel electrophoresis was performed using a QIAxcel Advanced capillary electrophoresis system (Qiagen, Hilden, Germany).

### 2.5. Immunofluorescence

Freshly dissected embryonic lungs were washed in sterile PBS (2 × 5 min), fixed in 4% PFA for 1 h (E14.5), 2 h. (E15.5), or 4 h. (E18.5) on ice, and then washed again (3 × 5 min.). Lungs were then dehydrated by successive washes in a graded ethanol series (30%, 50%, 70%, 100%, 100%) for 5 min. each, and stored in 100% ethanol at –20 °C. Samples were then paraffin embedded, sectioned to a thickness of 4–5 µm, and prepared for primary and secondary antibody staining as previously described [13] (See Table S1 for antibody details and dilutions).

### 2.6. RNA Lsolation and RT-qPCR

Total RNA was isolated as previously described [13]. Up to 1 µg of total RNA was reverse transcribed using the QuantiTect Reverse Transcription Kit (Qiagen, Hilden, Germany), following the manufacturer’s instructions. 

qPCR primers were designed to amplify mature mRNA using NCBI’s primer-BLAST option (https://www.ncbi.nlm.nih.gov/tools/primer-blast/) (last accessed, 29-06-2019), and expected product sizes were confirmed by PCR-based gel electrophoresis (see Table S2 for primer sequences). qPCR reaction mixtures were set up using the PowerUp SYBR Green Master Mix (Thermo Fisher, Schwerte, Germany), with a final volume of 20 µL for each reaction, according to the manufacturer’s instructions. Samples were run with three technical replicates on a LightCycler 480II (Roche, Mannheim, Germany) using the following protocol: UDG activation at 50 °C for 2 min; DNA polymerase activation at 95 °C for 2 min; and 40 cycles of denaturation at 95 °C for 15 s, annealing at 60 °C for 15 s, and extension at 72 °C for 1 min. To validate amplification specificity, a dissociation step was also included for each sample. Threshold cycles (Ct) were calculated and used to assess relative expression (∆Ct) using mouse *Hprt* as the reference gene.

### 2.7. Microarray

Differential gene expression was assessed using microarray analysis following the T7-protocol (detailed in M. R. Jones, Dilai, et al., 2019). Briefly, purified total RNA was amplified and Cy3-labeled using the LIRAK kit (Agilent Technologies, Waldbronn, Germany) following the kit instructions. Per reaction, 200 ng of total RNA was used. The Cy3-labeled RNA was hybridized overnight to 8 × 60 K 60 mer oligonucleotide spotted microarray slides (Agilent Technologies, design ID 028005). Slides were scanned at 2 µm/pixel resolution using the InnoScan is900 (Innopsys, Carbonne, France). Image analysis was performed with Mapix 6.5.0 software, and calculated values for all spots were saved as GenePix results files. Stored data were evaluated using R software (version 3.3.2) [19] and the limma package (version 3.30.13) [20] from BioConductor (open source, non-commercial software). Gene annotation was supplemented by NCBI gene IDs via biomaRt (last accessed 08-03-2018).

### 2.8. Microarray Expression Analysis

Mean spot signals were background corrected with an offset of 1 using the NormExp procedure on the negative control spots. The logarithms of the background-corrected values were quantile-normalized. The normalized values were then averaged for replicate spots per array. From different probes addressing the same NCBI gene ID, the probe showing the maximum average signal intensity over the samples was used in subsequent analyses. Genes were ranked for differential expression using an unpaired two-tailed Student’s t-test on a moderated t-statistic, and heatmaps were generated displaying genes according to descending p-values. Gene set tests were done on the ranks of the t-values, using the function ‘geneSetTest’ in the limma package from BioConductor. The number of independent samples (n) can be found in the figures. Gene sets were either user defined or, for pathway analyses, were based on the KEGG database (last accessed 08-03-2018). The data from the microarray experiments have been deposited in the NCBI’s gene expression omnibus (GEO accession GSE115876). 

### 2.9. FACS

Whole lungs were isolated in ice-cold Hank’s balanced salt solution (HBSS). Lungs were finely chopped with a sterile razor blade on a glass plate. The tissue was then added to a falcon tube and digested in 0.5% collagenase at 37 °C for 45 mins., with constant mixing. Single-cell suspensions were made by successively flushing the samples through 18 g, 20 g and 24 g grade needles and then filtering the samples through 70 µm and 40 µm nylon strainers. The cell suspensions were diluted with 5 mL HBSS and centrifuged at 12000 rpm for 5 min. and the supernatant was discarded. The pellet was resuspended in 10 µL blocking buffer and the following antibodies were added: 488-CD31 (1:50); FITC-CD45 (1:50); Apc Cy7 EpCam (1:50); Apc-Podoplanin (Pdpn) (1:20); and Apc Isotype Control (1:20) (all from Biolegend), for 20 min. at 4 °C. The samples were washed 2× with 100 µL FACS buffer and centrifuged at 12,000 rpm for 5 min. at 4 °C. The supernatant was discarded. The pellet was resuspended in 100 µL FACS buffer. Cell sorting and isolation were performed using the FACSAria™ III (BD Biosciences, Heidelberg, Germany) cell sorter. Alveolar epithelial cells were identified as CD45-/CD31-/Epcam+, AT1 cells as CD45-/CD31-/Epcam+/Pdpn+, AT2 as CD45-/CD31-/Epcam+/Pdpn-, and mesenchymal cells as CD45-/CD31-/Epcam-. Cells were sorted through a flow chamber with a 100-μm nozzle tip under 25 psi sheath fluid pressure. Isolated cells were used for RNA isolation. As a main criterion for gating, we used the settings to capture 98% of the cells in the isotype control and then we applied these gating conditions to the stained cells.

### 2.10. Gene Expression Patterns

To assess the expression patterns of genes in E14.5 lungs, the online database ‘Genepaint’, which contains in situ hybridization data for many genes, was used (https://gp3.mpg.de/) (last accessed 11-09-2019). Each of the genes significantly downregulated in our E14.5 + 9 h experiment was entered into genepaint. The whole embryo section displaying the clearest gene expression in the lung was chosen for the figure. 

### 2.11. Proliferation and Apoptosis

Proliferation was assessed using either antibody staining against Ki67 (see Table S1 for antibody information and dilution) or using the Click-iT EdU Imaging Kit (Invitrogen, Schwerte, Germany) according to the manufacturer’s instructions. For the EdU experiments, EdU was injected (i.p.) two h. before pregnant females were sacrificed (Dosage: 0.005 mg EdU/g mouse weight).

Ki67 fluorescence intensities were quantified using FIJI software (version 2.0.0-rc-68/1.52g). In the epithelial compartment of lung sections the average intensity of Ki67 was directly determined by quantifying each cell; however, in the mesenchyme, Ki67 intensity was assessed relative to DAPI intensity, and only in the distal regions of the samples, where DAPI intensity was comparable. EdU was quantified by determining the ratio of EdU-positive cells to total cells in each region of interest. Apoptosis was assessed on paraffin sections via the TdT-mediated dUTP Nick-End Labelling (TUNEL) assay using the DeadEnd Fluorometric TUNEL System (Promega, Walldorf, Germany) according to the manufacturer’s instructions. Apoptosis was quantified by determining the ratio of TUNEL-positive cells to total cells in each region of interest. 

### 2.12. Relative Gene Expression from qPCR Data

∆c_T_ values were calculated according to the following formula:

∆c_T_ = c_Treference_ − c_Tgene of interest_ (1)

Note, this equation accounts for the fact that c_T_ is proportional to the –Log of gene expression. ∆c_T_ is therefore positively related to the expression of the gene of interest. Significance was determined by unpaired two-tailed Student’s t-tests on the ∆c_T_ values, which can be assumed to be normally distributed. 

### 2.13. Statistics

Significance was determined by unpaired two-tailed Student’s t-tests, with p-values < 0.05 considered significant. Number of ‘n’ and observed significance levels are indicated either in the figures or in the figure legends.

## 3. Results

### 3.1. Identification of the Early Fgfr2b Transcriptomic Signature at E14.5 Supports a Primary Role for Fgf Signaling in Proliferation 

Our previously published paper on Fgfr2b signaling during the early pseudoglandular stage of lung development (E12.5), suggested that the primary Fgf ligand active at this stage was Fgf10 [13]. By inhibiting Fgf10/Fgfr2b signaling using our dominant negative soluble Fgfr2b mouse model, we were able to produce a strong phenotype within nine hours of inhibition. We found that Fgf10 primarily regulated distal epithelial cell–cell and cell–matrix adhesion and organization, as well as progenitor differentiation at this time. However, Fgfr2b signaling showed no discernable regulation of proliferation at this early stage.

**Figure 1:**
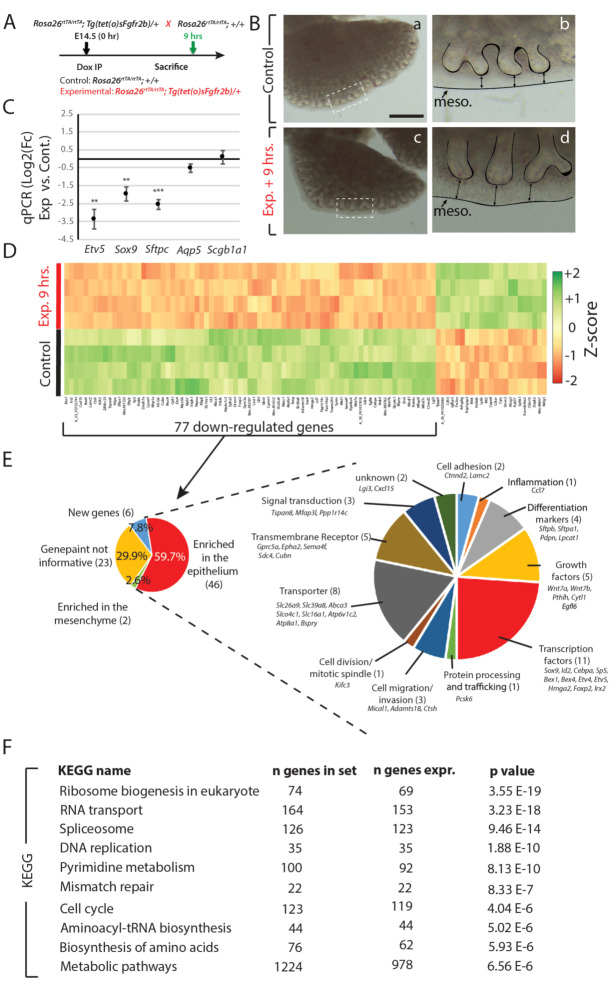
Transcriptomic effects of transient Fgfr2b signaling inhibition for 9 h in E14.5 lungs. (**A**) Experimental design: pregnant females carrying E14.5 experimental and littermate control embryos were injected with a single Dox-IP and sacrificed 9 h later. (**B**) Brightfield images of control (a, b) and experimental (c, d) lungs. Note the elongated epithelial tubes, reduced branching, and increased distance between the distal tips and mesothelium (black arrows) in the experimental lung compared to control. (Meso. = mesothelium). *Scale bar:* (a, c) 500 µm, (b, d) 125 µm. (**C**) qPCR analysis showing downregulation of *Etv5*, *Sox9*, and *Sftpc* in experimental vs. control lungs, while little change is observed in *Aqp5* and *Scgb1a1*. (*n* = 3; ** *p*-value < 0.01, *** *p*-value < 0.001). (**D**) Microarray expression data of the top 100 genes (according to the *p*-value) regulated in experimental vs. control lungs. (**E**) Gene ontology of the 77 downregulated genes from the heatmap in D. Genes were first grouped based on their region of enrichment in the lung according to expression data from ‘genepaint’. The 46 genes enriched in the epithelium, and thus considered to be direct targets of Fgfr2b signaling, were then grouped according to their primary biological functions. (**F**) KEGG pathway analyses of the entire microarray data showing the top 10 regulated pathways in the experiment, according to significance.

Using a similar approach at E14.5 (where other Fgfr2b ligands, especially Fgf1 and Fgf7, are increased compared to E12.5 levels [13], we inhibited Fgfr2b signaling for nine hours with a single Dox-IP (Figure 1A). The impacts of inhibition on the branching of the distal epithelium were evidenced in the elongation of distal buds, the thickening of the mesenchyme, and the reduced bud number in experimental lungs (Figure 1B). These gross morphological impacts were similar to those found at E12.5.

After extracting total RNA from these samples, we conducted qPCR analyses as well as a gene array. We determined that *Etv5*, a bona fide downstream target of Fgf10 signaling in the epithelium [21], was significantly downregulated in experimental compared to control samples, as were *Sox9* and *Sftpc*, which are canonical markers for distal epithelial cells and alveolar type II cells (AT2), respectively (Figure 1C). Markers for alveolar type I cells (AT1) and proximal epithelium, *Aqp5* and *Scgb1a1*, respectively, showed no significant regulation at this time point. These results suggest that Fgfr2b ligands act primarily on distal epithelial cells to maintain distal and AT2 progenitor status. Indeed, a gene-set analysis of AT1 and of AT2 signature genes from our E14.5 + 9 h. gene array showed a significant downregulation of AT2 markers in experimental lungs compared to controls, with little regulation on the AT1 signature (Figure S2A–C).

Next, we identified the top 100 genes regulated in the gene array (according to the p-value) (Figure 1D). These 100 genes comprised two groups of differential regulation, 77 of which were downregulated (and assumed, therefore, to be positively regulated by Fgfr2b signaling) and 23 of which were upregulated in experimental versus control lungs (See Figure S1 for an enlarged version of the heatmap). As at E12.5 [13], these current data were validated by confirming, from the array, the expected regulation of the well-established Fgf10-Fgfr2b-Etv4/5-Shh pathway. Both *Etv4* and *Etv5* were highly and significantly downregulated in our E14.5 array (Log_2_FC of –1.87 and –2.83, respectively), with a concomitant slight, yet significant downregulation of *Shh* (Log_2_FC = −0.51, *p*-value = 0.004) and its downstream mesenchymal target *Gli1* (Log_2_FC = −0.87, *p*-value = 0.00007). As a consequence, it is predicted that the *Fgf10* expression should increase with loss of Shh signaling, and indeed, that is what was found from the array (*Fgf10* Log_2_FC = 0.58, *p*-value = 0.0009). This evidence, along with the expected regulation of Wnt signaling (*Wnt7a* Log_2_FC = −1.04 and *Wnt7b* Log_2_FC = −1.55), and the downregulation of the distal epithelial transcription factor *Sox9* (Log_2_FC = −1.98), together with the predicted gross morphological changes observed, provide compelling evidence for the validity of our model to inhibit Fgfr2b signaling at this time point. 

**Figure 2:**
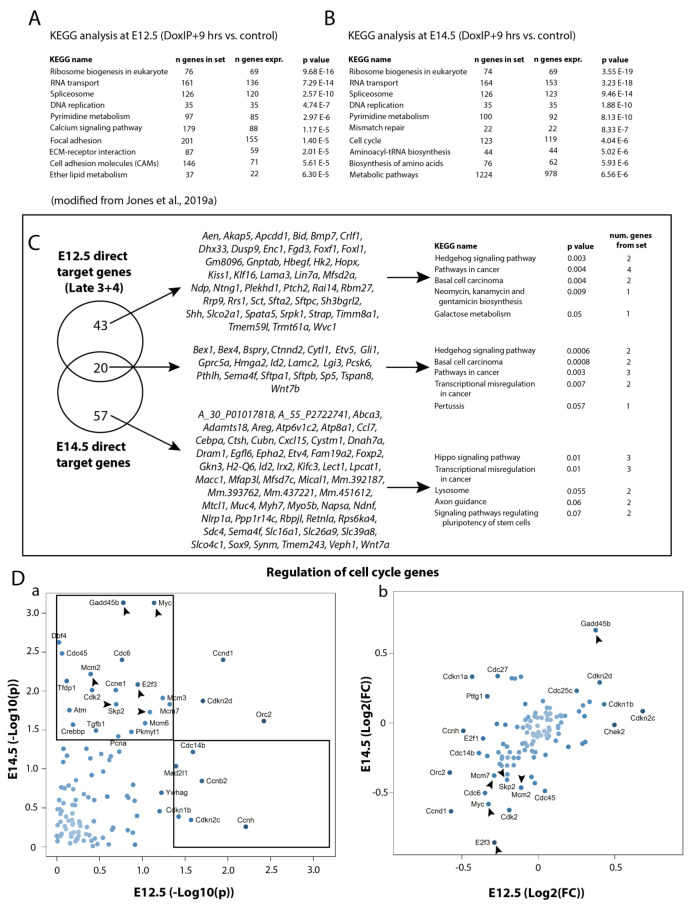
Comparison of the transcriptomic data between E12.5 and E14.5. (**A**) KEGG analysis at E12.5 and (**B**) at E14.5. (**C**) List of the genes which are specific to E12.5 and to E14.5, and which are shared among the two time points, along with the top five KEGG pathways regulated by each group of genes. (**D**) Scatterplots depicting the regulation, according to the p-value (a) and fold change (b) of cell cycle specific genes at E12.5 and at E14.5. Black arrows indicate significantly regulated genes specifically at E14.5. See text for details.

Using the online in situ hybridization database ‘Genepaint’, we found that 59.7% of the 77 downregulated genes are enriched in the epithelium and 2.6% in the mesenchyme, while 7.8% of the genes were not included in the database. The in situ data for the remaining 29.9% were uninformative (Figure 1E; Figure S3). Gene ontological analysis of the genes enriched in the epithelium found that they comprise 11 main biological processes: cell adhesion (*Ctnnd2* and *Lamc2*), inflammation (*Ccl7*), markers of differentiation (*Sftpb*, *Sftpa1*, *Pdpn*, and *Lpcat1*), growth factors (*Wnt7a*, *Wnt7b*, *Pthlh*, *Cytl1*, and *Egfl6*), transcription factors (*Sox9*, *Id2*, *Cebpa*, *Sp5*, *Bex1*, *Bex4*, *Etv4*, *Etv5*, *Hmga2*, *Foxp2*, and *Irx2*), protein processing and trafficking (*Pcsk6*), cell migration and invasion (*Mical1*, *Adamts18*, and *Ctsh*), cell division and mitotic spindle (*Kifc3*), transporter (*Slc26a9*, *Slc39a8*, *Abca3*, *Slco4c1*, *Slc16a1*, *Atp6v1c2*, *Atp8a1*, and *Bspry*), transmembrane receptor (*Gprc5a*, *Epha2*, *Sema4f*, *Sdc4*, and *Cubn*), and signal transduction (*Tspan8*, *Mfap3l*, and *Ppp1r14c*). Furthermore, we identified the top 10 KEGG pathways most significantly regulated by the genes in the array, and found that the most of them are critical for cellular proliferation (ribosome biogenesis, RNA transport, spliceosome, DNA replication, pyrimidine metabolism, cell cycle, aminoacyl-tRNA biosynthesis, biosynthesis of amino acids, and metabolic pathways) (Figure 1F). The top five of these E14.5 KEGG pathways were shared by the previously published top five E12.5 pathways (refer to GEO data, accession number GSE124157 from [13]); however, each of the remaining pathways was different between the two time points (compare Figure S3A and B). While at E12.5, pathways involved in cell–cell and cell–matrix adhesion were emphasized, at E14.5, pathways involved in the cell cycle and proliferation were more pronounced (see Figure 2A,B). This reflects the shift in the biological activity, from primarily a morphogenic to a proliferative effect, triggered by Fgfr2b ligands between early and mid-pseudoglandular stages. 

We further analyzed the top genes directly regulated by Fgfr2b signaling at E14.5 and E12.5 (according to the *p*-value). We found 43 genes specifically regulated at E12.5, 57 specifically regulated at E14.5, and 20 genes common among the two time points (Figure 2C). KEGG pathway analyses of these groups of genes revealed that pathways regulated at E12.5, such as hedgehog signaling and pathways related to cancer, were also regulated by the genes shared among E12.5 and E14.5; whereas the E14.5-specific genes regulated the hippo signaling pathway, lysosome, and axon guidance, and signaling pathways regulated the pluripotency of stem cells (Figure 2C).

Since none of these top target genes significantly regulated pathways related to cellular proliferation, we decided to plot the regulation of cell cycle-specific genes (as an indication of proliferation) from the entire E12.5 gene array compared to their regulation in the E14.5 array (Figure 2Da). Six cell cycle genes were specifically and significantly regulated at E12.5, 20 at E14.5, and three genes were shared between the two time points. Apart from the cyclin dependent kinase (*Cdk2*) and cell division control gene (*Cdc6*) significantly regulated specifically at E14.5, six other genes stand out (*E2f3, Myc, Mcm2, Mcm7, Skp2,* and *Gadd45b*) (see black arrows, Figure 2Da,b). Of these six genes, all except *Gadd45b* showed a downregulation upon Fgf ligands inhibition; *E2f3* and *Myc* are both transcription factors, the first regulating the cell cycle, and the second related to proliferation, apoptosis, and cellular transformation; *Mcm2* and *Mcm7* are members of the minichromosome maintenance complex which is needed for the initiation of DNA replication; and *Skp2* is necessary for DNA synthesis during replication. Interestingly, the sixth gene from this list, *Gadd45b*, was significantly upregulated at E14.5. This gene encodes a protein which inhibits cell growth and showed a similarly significant yet opposite regulation to *Myc*, suggesting that proliferation at this stage was not only affected by loss of promotion, but also by an increase in repression.

**Figure 3:**
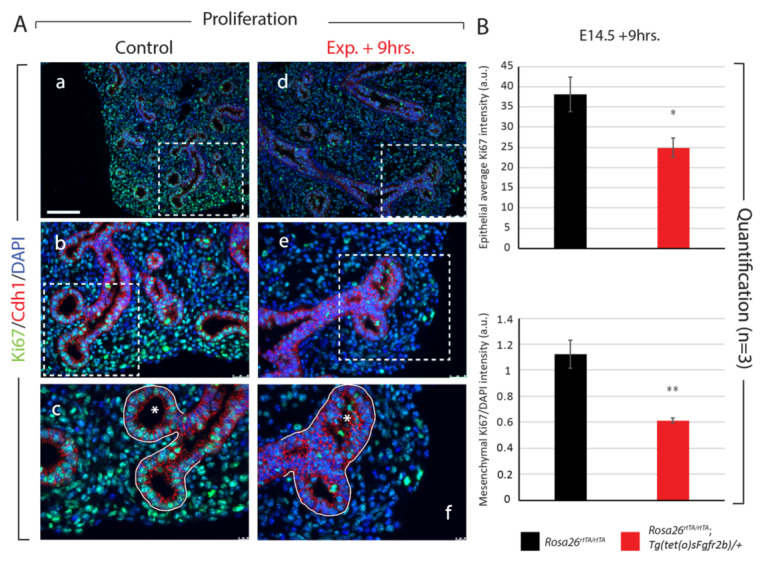
Analysis of proliferation in E14.5 lungs after 9 h of Fgfr2b inhibition. (**A**) Control (a–c) and experimental (d–f) lungs were stained for Ki67 and Cdh1 to assess proliferation in the epithelial and mesenchymal compartments (separated by the white line in c and f). Note the multilayered and disorganized epithelium invading the lumen of experimental lung buds (compare asterisks in c and f), which is a hallmark of Fgfr2b inhibition at E12.5 as well [13]. *Scale bar:* (a, d) 100 µm, (b, e) 50 µm, (c, f) 20 µm. (**B**) Quantification of Ki67 expression showing a significant decrease in proliferation in both the epithelial and mesenchymal compartments. (a.u. = arbitrary units) (* *p*-value < 0.05, ** *p*-value < 0.01).

To further investigate the regulation of proliferation at E14.5, we stained experimental and littermate control lungs nine hours after Fgfr2b inhibition, both for Ki67 and TUNEL, to assess proliferation and apoptosis, respectively (Figure 3). Ki67 intensity was significantly decreased in experimental lungs compared to controls, in both the distal epithelial and mesenchymal compartments (Figure 3A,B), whereas apoptosis was nearly absent in both control and experimental samples (data not shown). Of note is the observation of epithelial cells piling atop one another in a disorganized fashion in the lumens of experimental distal epithelial buds (compare Figure 3Ac,f). This is also a distinct phenotype seen at E12.5 + 9 h. inhibition [13].

Taken together, the results of Fgfr2b inhibition at E14.5 + 9 h. not only highlight the high correlation with the regulation of distal epithelial morphogenesis and differentiation at E12.5, but also emphasize the primary importance of Fgfr2b signaling in the proliferation of distal epithelial and mesenchymal cells at E14.5, likely through the cell cycle-specific genes identified from the array.

### 3.2. Inhibition of Fgfr2b Signaling for 24 h Drastically Decreases Proliferation and Impacts AT2 Differentiation

**Figure 4:**
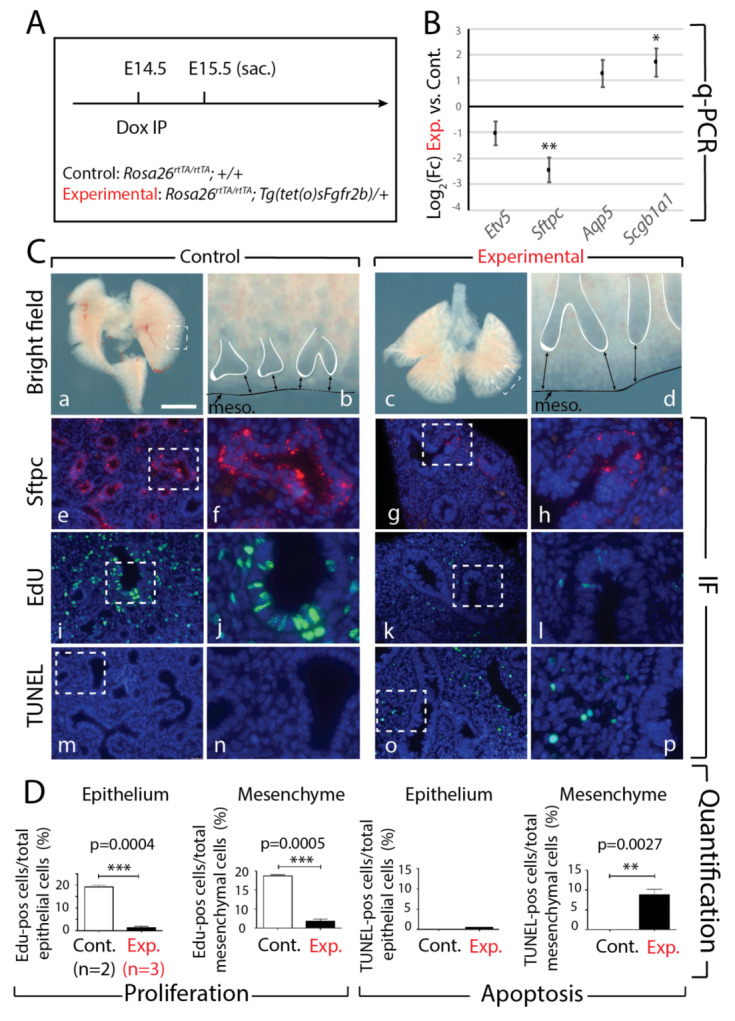
Effects of transient Fgfr2b signaling inhibition for 24 h in E14.5 lungs. (**A**) Experimental design: pregnant females carrying experimental and littermate control embryos were injected with a single Dox-IP and sacrificed 24 h later. (**B**) qPCR analysis showing downregulated expression of AT2 markers *Etv5* (not significant) and *Sftpc* (*n* = 3; ** *p* < 0.01), and upregulated expression of the AT1 marker *Aqp5* (not significant), and the proximal epithelial marker *Scgb1a1* (*n* = 3; * *p* < 0.05). (**C**) Brightfield (a–d) and immunofluorescent (e–p) images of control and experimental lungs. Note the elongated epithelial tubes, reduced distal tip branching, and increased distance between tips and mesothelium (black arrows) in experimental lungs compared to controls (d vs. b). Staining for Sftpc shows a decrease in the epithelium of experimental lungs (h vs. f), confirming the qPCR expression data. Staining for EdU and TUNEL to assess proliferation and apoptosis, respectively, showed decreased proliferation in both the epithelial and mesenchymal compartments of experimental lungs compared to controls (l vs. j), while apoptosis was increased only in the mesenchyme of experimental lungs (p vs. n). (meso. = mesothelium). *Scale bar:* (a, c) 750 µm, (b, d) 95 µm, (e, g, i, k, m, o) 75 µm, (f, h, j, l, n, p) 30 µm. (**D**) Quantification of EdU and TUNEL expressions in the epithelial and mesenchymal compartments of experimental vs. control lungs. (** *p*-value < 0.01, *** *p*-value < 0.0001).

To further assess the effects of short-term inhibition of Fgfr2b signaling on E14.5 lungs, we analyzed the phenotype of experimental lungs 24 h after Fgfr2b ligand inhibition (Figure 4A). Examination of the experimental and control lungs revealed impaired branching of the epithelium with the characteristic elongated distal branches and thicker mesenchyme (Figure 4Ca–d). qPCR analysis showed that expression of *Etv5* and *Sftpc* was reduced in experimental lungs compared to controls (Figure 4B). While the downregulation of *Sftpc* was significant, that of *Etv5* was not, which is in contrast to the highly significant downregulation of this gene after 9 h inhibition (see Figure 1C). This could be a consequence of the recovery of Fgfr2b signaling, 24 h after the single Dox-IP [17]. Furthermore, *Aqp5* and *Scgb1a1*, markers for AT1 cells and proximal epithelium, respectively, tended toward upregulation in experimental compared to control lungs. 

The downregulation of *Sftpc* in experimental lungs suggested regulation of the AT2 lineage. Indeed, impacted AT2 differentiation was confirmed by Sftpc immunofluorescence staining; while control lungs showed a robust and strong Sftpc expression in distal epithelium (Figure 4Ce,f), the expression in experimental lungs was clearly reduced (Figure 4Cg,h). Finally, analysis of proliferation and apoptosis, using EdU and TUNEL immunofluorescence, respectively, demonstrated a drastic reduction in proliferative cells, along with an increase in apoptotic cells, in experimental compared to control lungs (Figure 4Ci–l). Our quantification confirmed decreased proliferation in both the epithelium (*p* < 0.001) and the mesenchyme (*p* < 0.001), while cell death was increased in the mesenchyme (*p* = 0.0027) but not in the epithelium of experimental lungs (Figure 4D).

These results demonstrate that the impacts of inhibiting Fgfr2b signaling at E14.5 for 24 h. lead to effects on branching, proliferation, and progenitor cell differentiation similar to those found after 9 h., albeit, much more pronounced and drastic. The primary role of Fgfr2b signaling in proliferation was also confirmed at the 24-h time point; neither epithelial nor mesenchymal cells in experimental lungs regained proliferative capacity, while the number of apoptotic cells significantly increased in the mesenchyme.

### 3.3. Continuous Inhibition of Fgfr2b Signaling from E14.5 to E18.5 Leads to Impaired AT1 and AT2 Formation 

**Figure 5:**
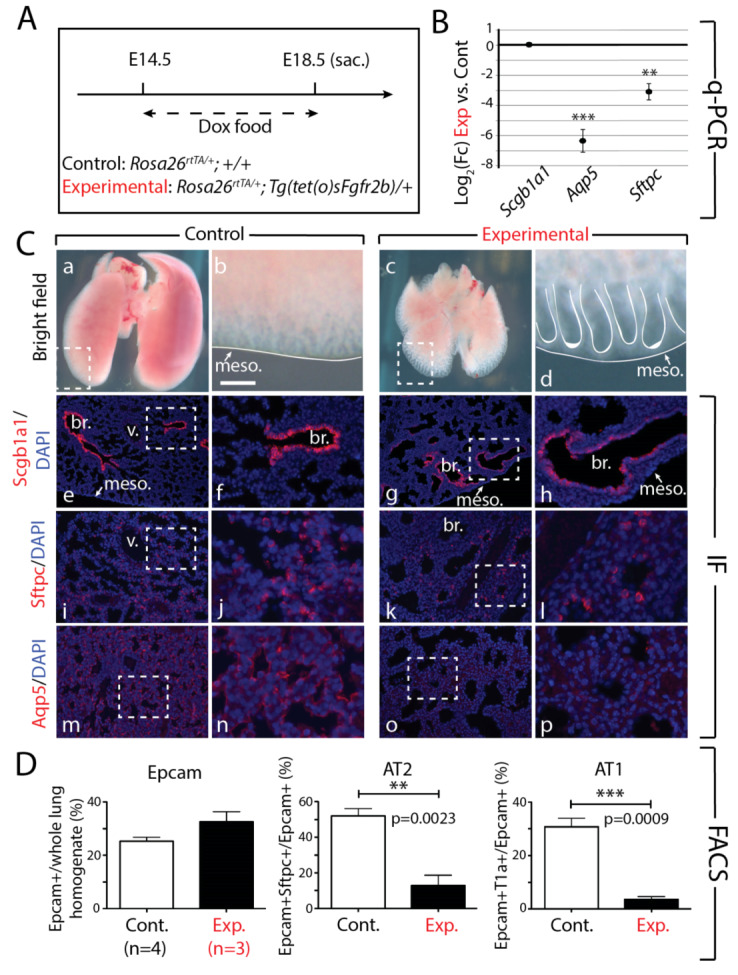
Continuous inhibition of Fgfr2b signaling from E14.5 to E18.5 leads to impaired AT1 and AT2 formation. (**A**) Experimental design: pregnant females carrying experimental and littermate control embryos were fed Dox food continuously for four days before being sacrificed at E18.5. (**B**) qPCR analysis showing significant downregulation of the AT1 marker *Aqp5* and the AT2 marker *Sftpc* in experimental versus control lungs. *Scgb1a1* shows no change in expression. (*n* = 3; ** *p*-value < 0.01, *** *p*-value < 0.001). (**C**) Brightfield (a–d) and immunofluorescent (e–p) images of control and experimental lungs. Note the smaller overall size and the dilated epithelial branches of experimental lungs compared to controls (c and d vs. a and b). Staining for Scgb1a1 shows a clear boundary between Scgb1a1+ proximal and Scgb1a1-negative distal epithelium in control lungs (e and f), whereas an ectopic distribution is seen in experimental lungs (g and h), with Scgb1a1+ cells being found in distal regions of the lung. Staining for Sftpc (i-l) and Aqp5 (m-p) reveal a downregulation of these proteins in experimental compared to control lungs, confirming the downregulation of these genes seen in the qPCR analysis (B). (meso. = mesothelium, br. = bronchus, v. = vessel). *Scale bar:* (a, c) 750 µm, (b, d) 95 µm, (e, g, i, k, m, o) 150 µm, (f, h, j, l, n, p) 30 µm. (**D**) FACS-based quantification shows no significant difference in the proportion of Epcam+ (epithelial) cells relative to the total (CD45-CD31-) lung cells in experimental versus control lungs, but a significant decrease in the proportion of AT2 (Epcam+ Sftpc+ from total Epcam+) (** *p*-value < 0.01) and AT1 (Epcam+T1alpha+ from total Epcam+) (*** *p*-value < 0.001) cells in experimental versus control lungs.

Next, we assessed the impacts of continuous Fgfr2b inhibition from E14.5 to E18.5 by feeding pregnant mice carrying experimental and littermate controls a diet of Dox food during this time (Figure 5A). Examination of the experimental and control lungs revealed abnormal lung development, with delayed growth resulting in noticeably smaller lungs, as well as dilated epithelial end buds, in experimental lungs compared to controls (Figure 5Ca–d). Immunofluorescence for the proximal club cell marker Scgb1a1 indicated that the Scgb1a1+ cells extended to the distal periphery of the lung in experimental vs. control lungs (Figure 5Ce–h). We also observed a severe reduction in Sftpc+ cells, as well as a complete absence of Aqp5+ cells in experimental lungs (Figure 5Ci–p). These findings were supported by qPCR analyses from RNA isolated from whole lungs, which showed a significant reduction in *Sftpc* and *Aqp5* expression in experimental vs. control lungs; however, no change was seen in *Scgb1a1* expression (Figure 5B). These results suggest that Fgfr2b signaling from E14.5 to E18.5 primarily affects AT1 and AT2 differentiation, while avoiding directly targeting the proximal lineage. 

Such a decrease in the numbers of AT1 and AT2 cells was also validated by flow cytometry (Figure 5D). The ratio of Epcam+ cells (Epcam, or the epithelial cell adhesion molecule, is ubiquitously expressed in the epithelium) to the whole lung homogenate did not change between experimental and control lungs, which is likely a consequence of arrested proliferation in both the epithelial and mesenchymal compartments of experimental lungs, thus resulting in smaller lungs overall. However, a clear reduction in the ratios of AT1 and AT2 (to the total number of Epcam+ cells) was observed (*p* = 0023 and *p* = 0.0009 for the ratio of AT2 and AT1 cells over Epcam in experimental vs. control lungs, respectively, Figure 5D). This result indicates that the alveolar lineage was completed aborted in experimental lungs, which corresponds well with the expansion of Scgb1a1+ cells into the distal epithelium (Figure 5Cg,h).

Taken together, results from the continuous long-term inhibition of Fgfr2b signaling beginning in E14.5 lungs suggest that Fgfr2b signaling, while promoting lung growth, also directly targets the alveolar lineage. In the absence of Fgfr2b signaling from E14.5 onward, both the AT1 and AT2 cells fail to form.

### 3.4. Transient Inhibition of Fgfr2b at E14.5 Leads to Irreversible Developmental Damages at E18.5

**Figure 6:**
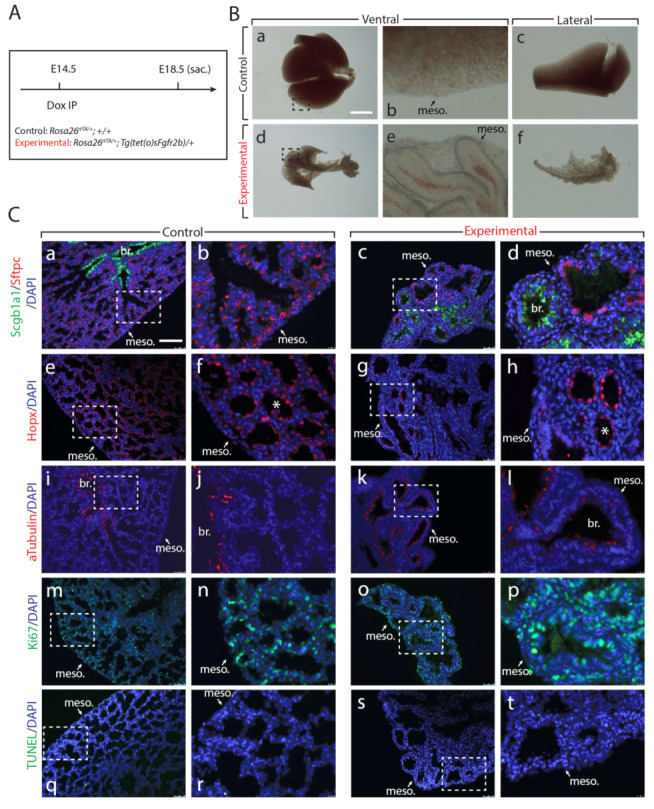
Experimental lungs are developmentally arrested after transient Fgfr2b signaling inhibition at E14.5. (**A**) Experimental design: pregnant females carrying experimental and littermate control embryos were injected with a single Dox-IP at E14.5, and embryos were harvested at E18.5. (**B**) Brightfield images of the ventral side of the whole lung in control (a, b) and experimental (d, e) samples, as well as the lateral perspective of the accessory lobe (c and f). Apart from the drastic difference in overall lung size between experimental and control lungs (d vs. a), notice the complete failure of the experimental epithelium to form the numerous branches and alveoli seen in the control, as well as the reduced mesenchyme and thickness of the lobes seen in the experimental lungs (f vs. c). (meso. = mesothelium). *Scale bar:* (a, d) 1.5 mm, (b, e) 95 µm, (c, f) 375 µm. (**C**) Immunofluorescent images (a-t) for control and experimental lungs. Antibody staining for AT1 (Hopx) and AT2 (Sftpc) cells suggests an overall reduction in the number of these cells in experimental compared to control lungs (compare c and g to a and e). Furthermore, in the case of Hopx staining in the experimental lungs (see g and h), there are cells with a similar expression pattern to the AT1 cells seen in controls, but also epithelial cells displaying a sparse, much reduced expression pattern (compare cells in the structure labeled by asterisks in h vs. f). Staining for the proximal epithelial markers Scgb1a1 and acetylated alpha-Tubulin reveals ectopic and distal expression in experimental compared to control lungs (compare d and l to b and j). Finally, staining for Ki67 (m–p) and TUNEL (q–t) to assess proliferation and apoptosis, respectively, shows that, in general, there is little to no impact on proliferative processes in either the epithelial or mesenchymal compartments. (meso. = mesothelium, br. = bronchus). *Scale bar:* (a, c, e, g, i, k, m, o, q, s) 125 µm, (b, d, f, h, j, l, n, p, r, t) 25 µm.

Given the strong phenotypic effects of a single Dox-IP after 9 and 24 h in experimental lungs, we were interested in investigating to what extent E14.5 lungs could recover from a single Dox-IP later in development. We therefore injected pregnant females at E14.5 and assessed experimental and littermate control embryos at E18.5 (Figure 6A). We previously showed that a single Dox-IP leads to the recombination of soluble Fgfr2b shortly thereafter, with levels of soluble Fgfr2b peaking around six hours before returning to pre-injection levels after 24 h [17]. Thus, a single Dox-IP in our mouse model leads to approximately 24 h of Fgfr2b signaling inhibition. 

Remarkably, experimental lungs assessed at E18.5 showed a much more drastic phenotype than experimental lungs from our continuous long-term inhibition of Fgfr2b (compare Figure 6B with Figure 5C). We propose that this is likely a consequence of the route of administration of the doxycycline between the two experiments (food vs. IP). An IP injection delivers to the system a relatively high dose of doxycycline in a short time, while doxycycline from food accumulates in the system gradually and over a longer period of time. Whereas experimental lungs from the continuous inhibition experiment were slightly smaller than control lungs, exhibiting reduced branching along with elongated epithelial tubes, the experimental lungs of the transient inhibition experiment were drastically different from littermate controls; these lungs were much smaller than controls, showed very long spaghetti-like non-branched epithelial tubes, and almost a complete absence of the mesenchyme in addition to reduced thickness of the lobes when viewed laterally (Figure 6Ba–f). 

Immunofluorescent stains for Sftpc and Hopx, markers for AT2 and AT1 cells, respectively, revealed the impacts of transient Fgfr2b inhibition on both lineages (Figure 6Ca–h). While in control lungs Sftpc and Hopx were robustly expressed in distal alveolar sacs, expression of these markers in experimental lungs was clearly decreased and of varying robustness, especially the Hopx protein (compare Hopx expression by the asterisks in Figure 6Ch). Staining for the proximal club cell marker Scgb1a1 also revealed the variable and disorganized distribution of proximal and distal epithelial lineages in experimental lungs (compare Figure 6Ca–d). While a clear demarcation between proximal and distal epithelium existed in control lungs, there was no clear demarcation in experimental lungs, with Scgb1a1 being expressed in both proximally and distally located epithelium (of interest, however, is the observation that Scgb1a1 expression did not colocalize with that of Sftpc, suggesting that individual epithelial cells had adopted either a proximal or distal cell fate, respectively). 

We also stained for other markers of proximal cell types. For example, acetylated alpha-Tubulin, a marker for ciliated cells, further confirmed the atypical expression of the proximal epithelial lineage in the distal epithelium of the experimental lungs (Figure 6Ci–l). While ciliated cells lined the proximal conducting airways of control lungs, they were scattered throughout the proximally and distally located epithelium of experimental lungs. 

Finally, to determine whether the drastic phenotype seen in experimental lungs was a consequence of aberrant proliferation, we stained control and experimental lungs for Ki67 and for TUNEL to assess proliferation and apoptosis, respectively (Figure 6Cm–t). The experimental lung showed a significant number of Ki67+ cells and did not appear different compared to the control lung. Apoptosis was not differentially regulated between control and experimental lungs. We therefore conclude that transient inhibition of Fgfr2b signaling, apart from completely arresting the gross morphological development of the lung, leads to the near total failure of the distal epithelium to form. In addition, the proximal epithelium, along with the remaining mesenchyme, appears to proliferate normally after transient Fgfr2b signaling inhibition.

## 4. Discussion 

In this paper, we report the role of Fgfr2b signaling in epithelial cells during mid-pseudoglandular lung development in E14.5 lungs. By using a dominant negative inducible model to inhibit Fgfr2b signaling, we identified the effects of transient inhibition of Fgfr2b ligands after 9 h., 24 h., and after 4 days, as well as continuous inhibition for 4 days. Macroscopically, the impacts of inhibiting Fgfr2b ligands at E14.5 are similar to those seen during early pseudoglandular development at E12.5 [13,21]. However, the primary biological activity of Fgfr2b signaling shifts from the regulation of branching morphogenesis (focal adhesion, regulation of actin cytoskeleton, cell to cell adhesion) seen at E12.5 to cellular proliferation and differentiation at E14.5. 

Our investigations during early (E12.5) and mid-(E14.5) pseudoglandular lung development highlight the shifting primary biological effects and direct transcriptomic targets of Fgfr2b signaling during this stage. For example, while the direct transcriptomic targets of Fgfr2b signaling at the two time points overlap remarkably (see Figure 2B), the most significantly regulated genes at E14.5 are not the same as those at E12.5. This difference is captured in the KEGG pathways regulated at each time point, with pathways related to the cell–cell and cell–extracellular matrix adhesion being significantly regulated at E12.5, and pathways related to cellular proliferation being most significant at E14.5. These differences translate at the cellular and morphological levels as well: inhibition of Fgfr2b ligands at E12.5 for 9 h. produced clear and drastic effects on branching morphogenesis without affecting proliferation or apoptosis, while inhibition of Fgfr2b ligands at E14.5 for 9 h. did lead to significant regulation of cellular proliferation, in both the epithelium and the mesenchyme (Figure 3). 

It is generally thought that stages of mammalian lung development are comparable among species [4]. Rodents, in particular, have been extensively used to study the basics of lung development, as well as models for human lung diseases and disorders. However, the success of translating these findings to the human context has been limited [22]. This is likely partly a consequence of differences in the molecular mechanisms driving each stage of development, when comparing species. For example, Fgf-signaling plays one of the most critical roles throughout lung development, and has been extensively studied in the mouse, with more recent studies focusing on humans [23,24]. In the mouse, Fgf10 and its cognate receptor Fgfr2b are essential for branching morphogenesis during the pseudoglandular stage of development, whereas in humans, FGF10 appears to be dispensable during this period, becoming more critical during the later canalicular stage [24].

One way to reconcile these data is the possibility that what we consider to be the pseudoglandular stage in humans (5 to 17 weeks) and in mice (E9.5 to E16.5) is not completely accurate. A big variable is the so-called embryonic stage, which is poorly characterized from a cellular and molecular point of view and which has been proposed to be from E9.5 to E12 in mice. This early stage is also characterized by a process of branching and it could therefore be proposed that the same cellular and molecular mechanisms are at work between the embryonic stage and the early pseudoglandular stage (E12.5) in rodents. Interestingly, unpublished results from our laboratory, i.e., transiently inhibiting Fgfr2b signaling at E9.5 to E10.5, show that the lungs are functional at birth (Danopoulos and Bellusci, as well as [17]) and display minor defects compared to the inhibition of Fgfr2b signaling at E10.5, which completely abolishes the branching process. Interestingly, in line with the known role of Fgf10/Fgfr2b signaling in the induction of the apical ectodermal ridge during limb development, inhibition at E9.5 abolishes limb development and therefore serves as an internal control for the validation of soluble Fgfr2b functional expression [17]. These results suggest that indeed the biological activities triggered by Fgfr2b signaling during the embryonic phase (which is likely restricted to E9.5–E10.5 in mice) are different compared to the ones during the early pseudoglandular stages. In addition, our work shows that this is also the case between the early and mid-pseudoglandular stages. Therefore, the same could apply to what we consider to be the human equivalent of the pseudoglandular stages. Our results showing that recombinant FGF10 does not trigger increased branching in 10–12-week human embryonic lungs could simply be due to the fact that these lungs are still at the so-called embryonic stage, which we suggest to be FGF10-independent in the context of the branching process [23]. 

Another important aspect of our work is linked to the formation of the alveolar epithelia lineage. It was previously thought that mature AT1 and AT2 cells arise from a common progenitor at E14.5 called the bipotent progenitor (BP). BPs express a combination of genes which are specific to both mature AT1 and AT2 cells [25]. More recently, this model has been challenged; lineage tracing of BPs using a dual recombinase transgenic system showed that these cells do not contribute significantly to the AT2 and AT1 lineages [26]. Instead, it was proposed that progenitors specific for these two lineages are already specified at the early pseudoglandular stages (E13.5). Our results indicate that one of the immediate effects of Fgfr2b signaling at E14.5 is to control the proper differentiation of AT2 progenitor cells while AT1 progenitors are not affected. However, long-term inhibition clearly also impacts the AT1 lineage. We therefore conclude that at E14.5 Fgfr2b, signaling is important for the formation of both the AT2 and the AT1 lineages. Further experiments using specific drivers for AT1 or AT2 progenitor cells should be used to delete Fgfr2b and evaluate the cell-autonomous impact of Fgfr2b signaling inhibition in these two alveolar epithelial lineages.

In conclusion, our work has assessed for the first time the impacts of Fgfr2b signaling inactivation on E14.5 lungs and has uncovered some of the convergent and divergent sets of biological activities and transcriptomic targets compared to the results obtained at E12.5. Our results suggest that stage-specific Fgfr2b signaling activities may be at play from the embryonic to the early to mid-pseudoglandular stages of lung development. Further studies will have to be conducted to elucidate the shifting roles of Fgfr2b signaling at successive stages (canalicular/saccular/alveolar) of lung development as well as during homeostasis and regeneration/repair after injury. As this paper demonstrates, more detailed studies within each stage of lung development are needed to better understand how Fgfr2b signaling mechanistically operates at the molecular and cellular levels to control lung organogenesis. 

## Supplementary Materials

The following are available online at https://www.mdpi.com/2073-4409/9/5/1274/s1, Figure S1: Enlarged heatmap from Figure 1D showing the top 100 regulated genes (according to p-value) from E14.5 + 9 h gene array. Figure S2: Experimental lungs (E14.5 + 9 h.) display impaired AT2, but not AT1, differentiation. Figure S3: Expression patterns of genes directly regulated after 9 h by Fgfr2b signaling in E14.5 lungs. Table S1: Primary and secondary antibodies. Table S2: qPCR primers.
