# Supplementary material for: Evidence for Overlapping and Distinct Biological Activities and Transcriptional Targets Triggered by Fibroblast Growth Factor Receptor 2b Signaling between Mid- and Early Pseudoglandular Stages of Mouse Lung Development
Source: Cells. 2020 May 21;9(5):1274. doi: 10.3390/cells9051274 (PMC7290466; doi:10.3390/cells9051274)
Supplement: Supplementary file 1 [file cells-09-01274-s001.pdf]

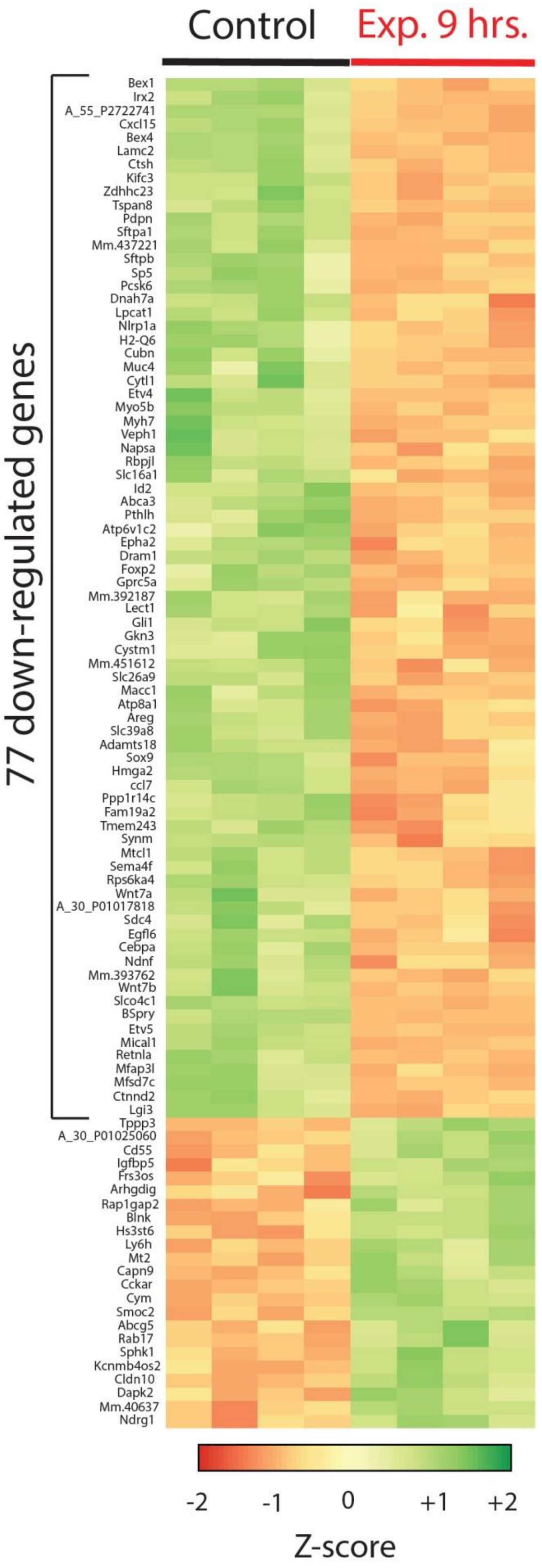

Figure S1: Enlarged heatmap from Figure 1D showing the top 100 regulated genes (according to p-value) from E14.5 + 9 hours gene array.

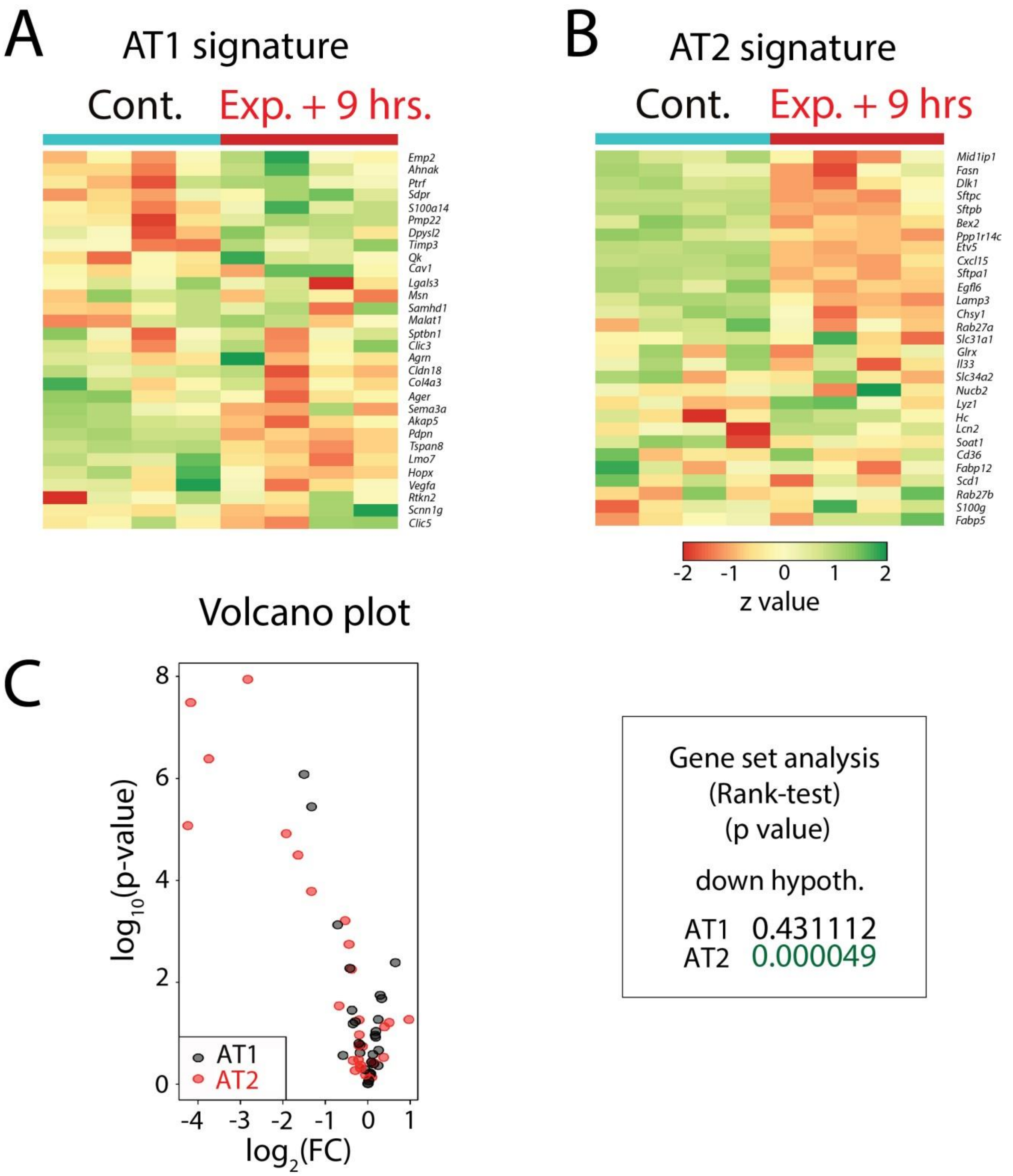

Figure S2: Experimental lungs (E14.5 + 9 hrs.) display impaired AT2, but not AT1, differentiation. (A) Heatmap for the AT1 and **(B)** AT2 gene signature using the E14.5 + 9 hrs. experimental and control lungs. **(C)** Corresponding volcano plot and **(D)** gene set analysis showing a significant downregulation of the AT2 (p=0.000049), but not the AT1, signature.

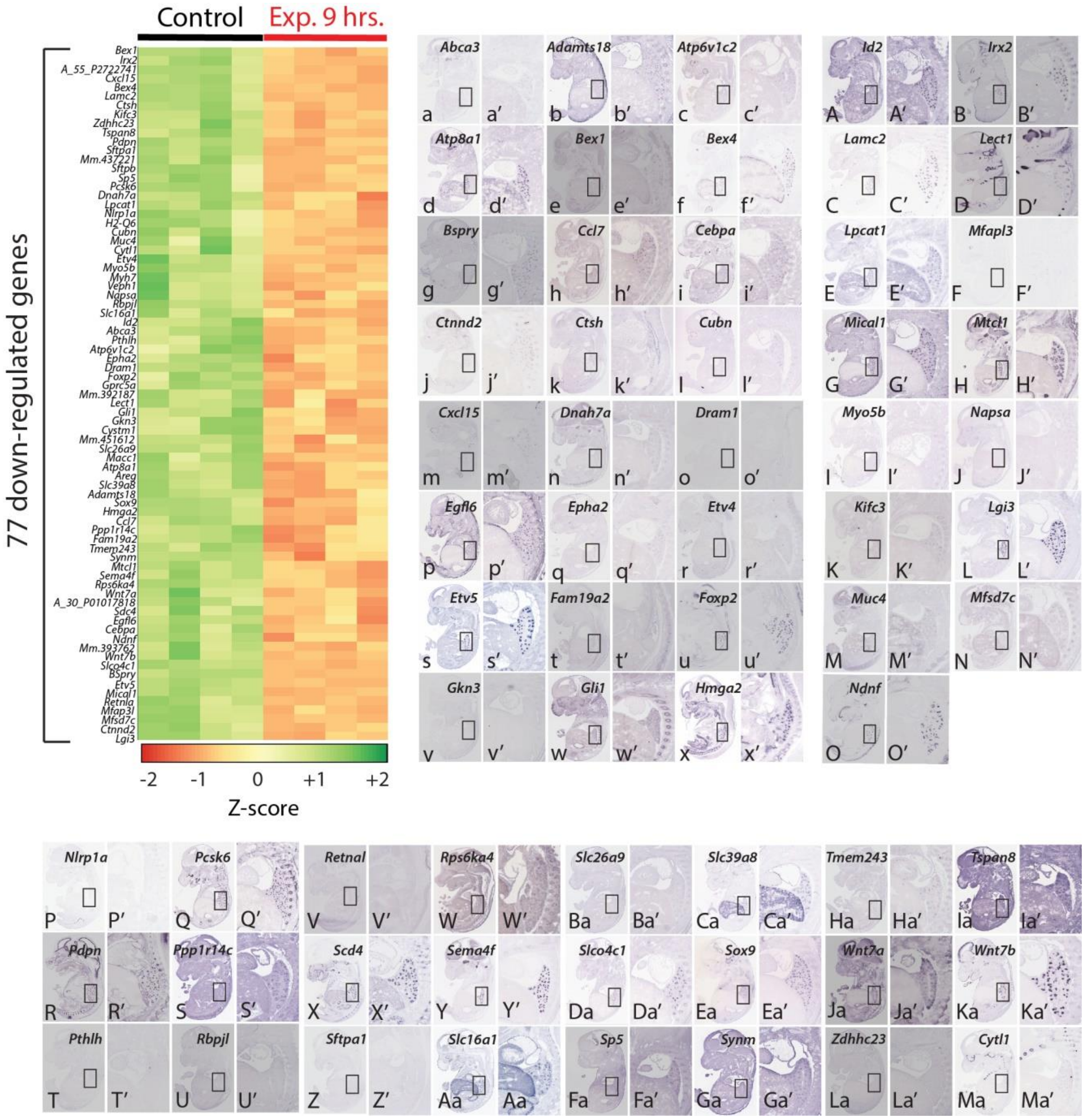

Figure S3: Expression patterns of genes directly regulated after 9 hours by Fgfr2b signaling in E14.5 lungs. (A) Heatmap for the 77 genes downregulated from the top 100 regulated genes, constituting the Fgfr2b ligands signature at E14.5. **(B)** Corresponding *in situ* hybridization expression patterns for the identified genes (from 'Genepaint').

Table S1: Primary and secondary antibodies

Antibody	Host	Dilution	Company (Cat. number)
Acetylated a-Tubulin	Mouse	1:1000	Sigma (T6793)
Aqp5	Rabbit	1:200	CalBiochem (178615)
Cdh1	Mouse	1:200	BD Biosciences (610181)
Hopx	Rabbit	1:200	Sigma (HPA030180)
Ki67	Rabbit	1:200	Cell Signaling (9129)
Scgb1a1	Goat	1:100	Santa Cruz (sc-9772)
Sftpc	Rabbit	1:500	Millipore (AB3786)

Alexa Fluor 555 anti-rabbit	Goat	1:500	Invitrogen (A21429)
Alexa Fluor 488 anti-goat	Donkey	1:500	Invitrogen (A11055)
Alexa Fluor 555 anti-mouse	Donkey	1:500	Invitrogen (A31570)
Alexa Fluor 568 anti-goat	Rabbit	1:500	Invitrogen (A11079)
Alexa Fluor 488 anti-rabbit	Goat	1:500	Invitrogen (A11034)

Table S2: qPCR primers

**Gene**	**Forward primer**	**Reverse primer**	**Expected product size**
Aqp5	5'-TAA CCT GGC CGT CAA TGC-3'	5'-GCC AGC TGG AAA GTC AAG AT-3'	84 bp
Etv5	5'-TAG CTG AAG CAC AAG TTC CTG A-3'	5'-GCA GCT CCC GTT TGA TCT TG-3'	105 bp
Hprt	5'-TCC TCC TCA GAC CGC TTT TT-3'	5'-ATC ATC GCT AAT CAC GAC GC-3'	82 bp
Scgb1a1	5'-GAT CGC CAT CAC AAT CAC TG-3'	5'-CAG ATG TCC GAA GAA GCT GA-3'	66 bp
Sftpc	5'-GGT CCT GAT GGA GAG TCC AC-3'	5'-GAT GAG AAG GCG TTT GAG GT-3'	94 bp
Sox9	5'-AGT CGG TGA AGA ACG GAC AA-3'	5'-CTG AGA TTG CCC AGA GTG C-3'	158 bp
